# Awareness of Keratoconus and Its Association With Eye Rubbing Among the Population in Aseer Province

**DOI:** 10.7759/cureus.41271

**Published:** 2023-07-02

**Authors:** Abdulrahman Alamri, Amal A Alrizqi, Amal A Aljohani, Danah A Alzahrani, Othman M Alassaf, Yazeed A Hamzi, Norah M Alharbi, Basil A Alharbi, Medhat Taha

**Affiliations:** 1 Department of Ophthalmology, College of Medicine, King Khalid University, Abha, SAU; 2 Department of Medicine and Surgery, Al-Qunfudah College of Medicine, Umm Al-Qura University, Alqunfudhah, SAU; 3 Department of Medicine and Surgery, College of Medicine, King Khalid University, Abha, SAU; 4 Department of Medicine and Surgery, College of Medicine, University of Hail, Hail, SAU; 5 Department of Medicine and Surgery, College of Medicine, Jazan University, Jazan, SAU; 6 Department of Medicine and Surgery, Unaizah College of Medicine and Medical Sciences, Qassim University, Qassim, SAU; 7 Department of Surgery, Ar Rass General Hospital, Ar Rass, SAU; 8 Department of Anatomy, Al-Qunfudhah Medical College, Umm Al-Qura University, Makkah, SAU; 9 Department of Anatomy and Embryology, Faculty of Medicine, Mansoura University, Mansoura, EGY

**Keywords:** allergy, asir, rubbing eye, awareness, keratoconus

## Abstract

Background: Keratoconus (KC), is a non-inflammatory, bilateral, asymmetrical, progressive disease characterized by ectasia, thinning, increasing corneal curvature, and loss of visual acuity. In Saudi Arabia, it was discovered that KC has been the main reason for corneal transplantation in the past 20 years. Eye rubbing is considered one of the most significant risk factors for KC based on available data.

Participants and methods: A cross-sectional study was conducted among adults in Aseer province, Saudi Arabia in 2023. Data were collected through a pre-designed online questionnaire consisting of 17 questions divided into three sections. The questionnaire was preceded by informed consent and insured to maintain the confidentiality of the data.

Results: A total of 498 respondents were included in this study. The majority of the study's participants were in the 18 to 25 age group and females made up the majority. The majority of participants had a university education. 41.6% of individuals reported experiencing an allergic disorder, 59.0% reported having knowledge about KC, and the overall participants’ awareness regarding KC was poor at 85.74%, with the highest percentage of good awareness (22.2%) detected among the 18-25 age group.

Conclusion: The study revealed a concerning lack of awareness about KC among the population in Aseer Province, Saudi Arabia. Additionally, a significant percentage of participants engaged in eye rubbing, a behavior associated with increased risk for KC. There is a need for increased awareness about KC and the importance of avoiding eye rubbing.

## Introduction

The condition known as keratoconus (KC), which was originally described in 1854, is a non-inflammatory, bilateral, asymmetrical, progressive disease marked by ectasia, thinning, increasing corneal curvature, and loss of visual acuity, especially in those with significant irregular astigmatism [1]. According to estimates, there are 1.38 cases of KC for every 1,000 persons [2]. It was discovered in Saudi Arabia that the main reason for corneal transplantation in the past 20 years was KC [3], The most significant risk factors for KC, as determined by the data, were eye rubbing, familial history of KC, allergy, asthma, and eczema [2].

Eye rubbing is a widespread behavior among people and happens both before sleep and upon awakening. It happens in reaction to mental stress, weariness, or ocular inflammation. Symptoms of allergies and eye dryness might cause people to massage their eyes. Eye rubbing is a frequent practice that happens spontaneously before sleep and upon awakening, as a response to ocular discomfort, weariness, and mental stress [4]. Chronic aberrant eye rubbing is a well-known risk factor for the development of KC [5]. The aim of this study was to assess the level of awareness about KC in Aseer Province and its relationship with eye rubbing.

## Materials and methods

A cross-sectional study was conducted from March to May 2023 among adults in Aseer Province, Saudi Arabia, to assess the level of awareness about KC. Ethical approval was obtained from the Research and Ethics Committee of King Khalid University, Abha, Saudi Arabia (approval number: ECM#2023-1105). The calculated sample size was based on the latest census in Aseer province (2,024,285). Using the Roasoft calculator with a 5% margin of error and 95% confidence interval, the estimated calculated sample size was 385 participants. However, the final sample size was 498 after data collection. The participants were randomly chosen, and the questionnaire was distributed using a self-administered Google form through a WhatsApp broadcast message (Meta Platforms, Inc., Menlo Park, California, United States) that contained the questionnaire, study rationale, and research objectives. Data were collected through a pre-designed online questionnaire from a prior study conducted in Jeddah, Saudi Arabia [6]. The questionnaire was translated into Arabic and back to English. The validity of the questionnaire was tested through a pilot study, the data from which were excluded from the main study. The questionnaire consisted of 17 questions and was divided into three sections, preceded by informed consent, and insured to maintain the confidentiality of the data. The first section included the socio-demographic profile, while the second section inquired about the medical and family history of eye diseases among study participants. The awareness and perception of KC and its relationship to eye rubbing were assessed in the third section.

Data analysis

The data collected was reviewed, coded, and inputted into IBM SPSS Statistics for Windows, Version 22.0 (Released 2013; IBM Corp., Armonk, New York, United States) after extraction. Statistical analyses were performed using the Pearson Chi-Square test, with statistical significance set at p<0.05. For awareness questions, each correct response was given a score of one point, and the total score was calculated. Poor awareness was defined as a score of less than 75%, and good awareness was defined as a score of at least 75%. Descriptive analysis was performed on all variables, including participants' biographical data, family history of KC, medical history of eye illnesses, and their source of knowledge regarding KC, based on frequency and percent distribution. Frequency tables and graphs were used to assess participants' awareness levels regarding KC and its risk factors and treatment methods, excluding the practice of eye rubbing. Cross-tabulation was used to determine the distribution of participants' awareness levels based on their personal data and practices.

## Results

The study questionnaire was completed by 498 individuals. Table 1 reveals that the age of the participants varied from 18 years to over 45 years, with the majority of the study's participants being in the 18 to 25 age group, which comprised 194 (39%) of the total participants. Females were the majority, constituting 309 (62.0%) of the participants. In terms of education level, 144 individuals (28.9%) had a high school education, 312 (62.7%) had a university education, and only 42 (8.4%) had primary education.

**Table 1:** Sociodemographic data of study participants

		Count	Column, N %
Gender	Male	189	38.0
	Female	309	62.0
Age Group	18-25	194	39.0
	26-35	132	26.5
	36-45	85	17.1
	Older than 45	87	17.5
Education Level	Primary education	42	8.4
	High school education	144	28.9
	University education or higher	312	62.7

Table 2 illustrates that out of the total participants, 207 individuals (41.6%) reported experiencing an allergic disorder. Nasal allergy was the most frequently reported allergy, with 98 (19.7%) of the participants indicating that they suffered from it. Skin allergy was the second most commonly reported allergy, with 64 (12.8%) participants reporting it, followed by eye allergy (62 participants, 12.4%) and chest allergy (61 participants, 12.2%).

**Table 2:** Medical and family history of eye diseases among study participants

		Count	Column N %
Had a history of allergy	Yes	207	41.6%
	No	291	58.4%
Type of allergy:			
Skin allergy		64	12.8%
Nasal allergy		98	19.7%
Chest allergy		61	12.2%
Eye allergy		62	12.4%
Gastrointestinal tract allergy		26	5.2%
Food and‎/or antibiotics allergy		2	0.4%
Urticaria		2	0.4%
Had a refractive error or any condition that affects the eye?	Yes	238	47.8%
	No	260	52.2%
What is the disorder?			
Myopia/hypermetropia		144	28.9%
Previous refractive surgery		44	8.8%
Keratoconus		20	4.0%
Astigmatism		8	1.6%
Prescription glasses		2	0.4%
Previous eye surgery		16	3.3%
Using contact lenses		41	8.2%
Amblyopia		19	3.8%
Others		11	2.2%
Family history of keratoconus	Yes	119	23.9%
	No	379	76.1%

When it comes to visual/eye disorders, 238 (47.8%) participants had a history of such conditions (Table 3). Refractive error was the most commonly reported disorder, with 28.9% of participants indicating that they had it. Previous refractive surgery was reported by 8.8% of the participants, while 8.2% reported using medical contact lenses, and 4% reported having KC. A family history of KC was reported by 119 (23.9%) participants.

**Table 3:** Awareness and perception of keratoconus among study participants

		Count	Column N %
Heard about keratoconus	Yes	294	59.0%
	No	204	41.0%
Source of information	Physician	71	22.2%
	Social media	72	22.5%
	Friends	73	22.8%
	Lectures/reading	104	32.5%
What is keratoconus?	Thinning of corneal thickness	134	26.9%
	Inflammation of the cornea	26	5.2%
	Increased corneal thickness	103	20.7%
	I do not know	235	47.2%
Is there a relationship between keratoconus and allergy?	Yes	136	27.3%
	No	49	9.8%
	I do not know	313	62.9%
Does keratoconus lead to visual impairment?	Yes	294	59.0%
	No	19	3.8%
	I do not know	185	37.1%
What is the treatment modalities of keratoconus			
Surgery		184	36.9%
Medical Contact Lenses		69	13.8%
Eyedrops		36	7.2%
Prescription glasses		75	15.0%
There are no treatments		19	3.8%
I do not know		241	48.3%
Frequent eye rubbing is	A habit that may leads to keratoconus	119	23.9%
	A habit that may harm the eye	165	33.1%
	It may cause allergy/itch	79	15.9%
	It's a safe habit	8	1.6%
	I do not know	127	25.5%

Out of all the study participants, 59.0% reported having knowledge about KC. The majority of them obtained their information through lectures/reading (32.5%), followed by friends (22.8%), social media (22.5%), and physicians (22.2%). Regarding their understanding of KC, 26.9% knew that it involves a decrease in corneal thickness, 27.3% believed that there is a relationship between KC and allergy, and 59% reported that it can lead to visual impairment.

When it comes to the treatment of KC, 36.9% of the study participants reported that surgery is a treatment option, 15.0% were aware of wearing medical glasses, and 13.8% mentioned medical contact lenses. In terms of the causes of KC, 23.9% of the participants agreed that frequent eye rubbing is a habit that may lead to KC, while 33.1% believed that it is generally a harmful habit. Additionally, 15.9% thought that it may cause allergy/itch, and 1.6% believed that it is a safe habit.

**Figure 1:**
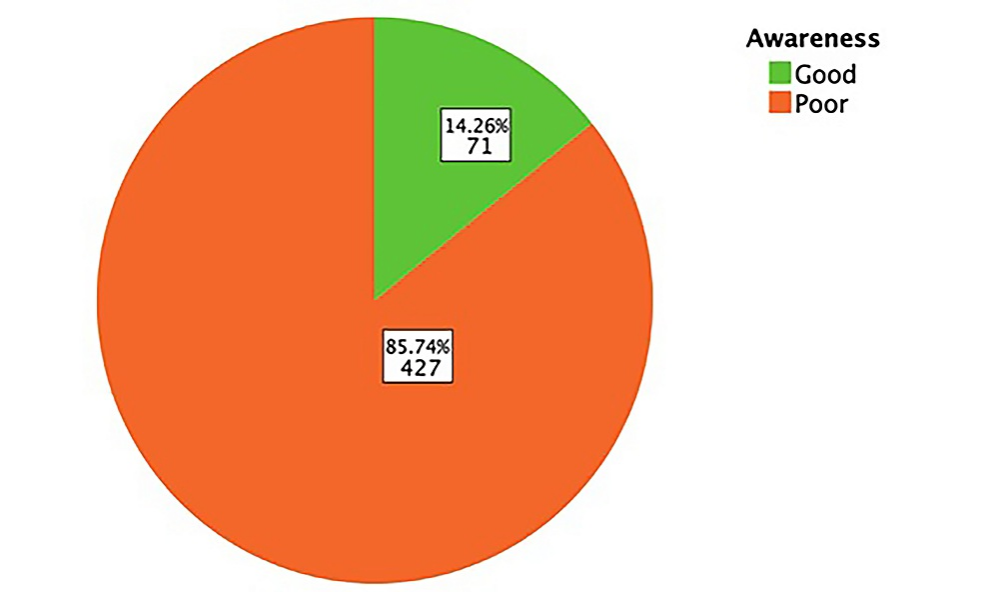
Overall awareness about keratoconus among study participants

The awareness levels among study participants regarding KC are illustrated in Figure 1. Out of the total participants, 427 individuals (85.74%) had poor awareness levels regarding KC, while only 72 (14.26%) had good awareness.

**Figure 2:**
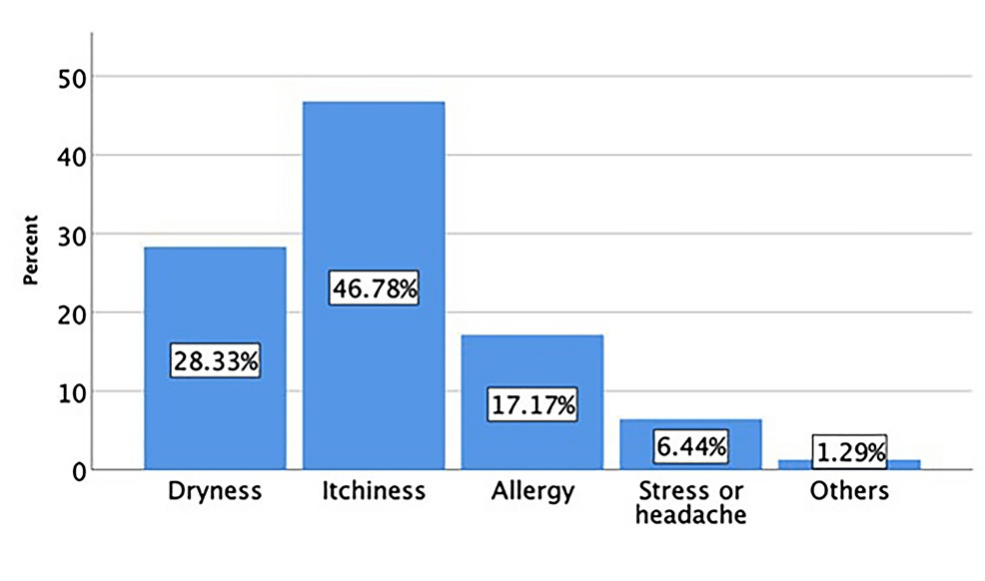
Causes for eye rubbing reported by study participants

The causes of eye rubbing reported by study participants are depicted in Figure 2. Out of all the participants, 617 individuals (80.4%) reported rubbing their eyes. The most commonly reported cause of eye rubbing was itchiness (46.78%), followed by dryness (28.33%), allergy (17.17%), stress or headache (6.44%), and other causes (1.29%).

Table 4 presents the overall awareness levels regarding KC stratified by the sociodemographic data and medical history of the participants. The age group was found to be statistically significant (P=0.001), with the highest percentage of good awareness (22.2%) detected among the 18-25 age group. Educational level was also found to be statistically significant (p=0.007), with participants who had a university education having a higher percentage of good awareness (17.9%) than those with other educational levels.

**Table 4:** Overall awareness of the participants regarding keratoconus according to their sociodemographic data and medical history *A p-value less than 0.05 was considered statistically significant

		Awareness				
		Good		Poor		
		Count	Row N %	Count	Row N %	p-Value Chi-Square
Gender	Male	23	12.2%	166	87.8%	0.279
	Female	48	15.5%	261	84.5%	
Age Group	18-25	43	22.2%	151	77.8%	0.001
	26-35	14	10.6%	118	89.4%	
	36-45	9	10.6%	76	89.4%	
	Older than 45	5	5.7%	82	94.3%	
Education Level	Primary education	2	4.8%	40	95.2%	0.007
	High school education	13	9.0%	131	91.0%	
	University education or higher	56	17.9%	256	82.1%	
Had a history of allergy	Yes	35	16.9%	172	83.1%	0.154
	No	36	12.4%	255	87.6%	
Had a refractive error or any condition that affects the eye	Yes	45	18.9%	193	81.1%	0.005
	No	26	10.0%	234	90.0%	
Family history of keratoconus	Yes	18	15.1%	101	84.9%	0.756
	No	53	14.0%	326	86.0%	
Did you rub your eyes regularly?	Yes	23	11.5%	177	88.5%	0.149
	No	48	16.1%	250	83.9%	

Good awareness was detected among 18.9% of participants with visual problems compared to 10% of those without visual problems, and this was statistically significant (p=0.005). However, 15.1% of participants with a family history of KC had good awareness regarding the disease compared to 14% of those without a family history, but this was not statistically significant (p=0.756). All other factors were not significantly associated with the participants' awareness levels.

## Discussion

The prevalence of KC, a bilateral and asymmetric progressive corneal thinning and steeping [7], was 138 per 100,000 people worldwide according to a recent meta-analysis [2]. However, the prevalence of KC varied, being as high as 5% in the Middle East [8]. In this study, the prevalence of KC among participants was high at 4%. The condition has well-described clinical indicators, but unless the anterior corneal topography is examined, the early stages may go undiagnosed [9]. The most prevalent primary ectasia is KC. It typically affects people in their second decade of life and crosses all racial and ethnic boundaries [10].

Progressive corneal protrusion and thinning, which cause uneven astigmatism and impair visual function, are characteristics of KC. There is still much to learn about the condition's pathogenesis and etiology [11]. The aim of this study was to assess the level of awareness about KC in Aseer Province and its relationship with eye rubbing.

The study's participants' level of knowledge on KC is extremely low; out of all participants, 85.74% had poor knowledge, while just 14.26% had high knowledge. On the other hand, a study conducted in Riyadh shows a more diverse distribution of knowledge levels where 38.4% of participants had a high level of knowledge, 31.3% had a moderate level, and 30.3% had a poor level of knowledge about KC [12]. These differences in knowledge levels between the two studies could be attributed to the differences in study populations, methods used to assess knowledge, or educational programs available in the respective locations. We found that people aged between 18-15, those with a high education level, and people with eye conditions have a higher awareness, although the findings of another study that assessed the awareness of KC among the general population in Saudi Arabia showed that the knowledge regarding KC was poor irrespective of the sociodemographic characteristics [13].

In our study, 23.9% of participants had a family history of KC. While only 7.7% had a family history of KC in a study conducted in Medina [1], which was consistent with the results of Dundee University Scottish study where 5% of patients reported a family history of KC [14].

KC was consistently associated with eye rubbing [15]. In this study, 45% of KC participants reported rubbing their eyes frequently. Also, the overall prevalence of eye rubbing among the participants is 40%, the majority were due to itching, followed by dryness and allergies. Although it was a high percentage, it was less than what they found in another study conducted in Jeddah, where 75.8% were rubbing their eyes, and for most of them (40.9%) the cause of rubbing was eye itching [6]. Prevention of KC could be achieved by avoiding eye rubbing and managing the causes [7].

In terms of allergies, systemic allergy and ocular allergy in particular were found to be one of the key risk factors associated with KC pathogenesis [16,17]. In this study, 41.6% of participants reported experiencing an allergic disorder. Nasal allergy was the most frequently reported allergy 19.7%. Skin allergy was the second most commonly reported allergy 12.8% followed by eye allergy 12.4% and chest allergy 12.2%. Whereas in Medina, 34.9% had a history of allergy, and eye allergy is the most reported type 39.1% [1]. In addition, in Dundee University Scottish study showed that 67% of participants had a medical history of allergy, with hay fever being the most frequent type 30% [14].

The severity and progression of the KC affect how it is managed. Typically, mild cases are managed with eyeglasses, moderate cases with contact lenses, and severe cases that cannot be treated with scleral contact lenses may need corneal surgery [7]. In our survey, 36.9% of participants believed that surgery is an optional therapy; 15.0% were aware of using medical glasses; 13.8% reported using medical lenses; 7.2% mentioned eye drops; and 3.8% believed there are no treatments.

In our study the most reported public source of information regarding KC was Lectures/ reading (32.5%) followed by friends (22.8%); however, a cross-sectional study that assessed the awareness level of general population regarding KC in Aseer region, Southern of Saudi Arabia found the most source of information are family and friends (29.2%) followed by internet (18%) [18].

This study has some limitations that should be taken into consideration. First, only educated participants who are accustomed to utilizing internet technologies and have internet connections would be able to complete the online, self-administered questionnaire; thus, the study ignored the illiterate and those who did not have internet access. Despite the previously mentioned limitations, this study is an initiative to highlight this common disease in the general population.

## Conclusions

In conclusion, the findings of this cross-sectional study revealed a concerning lack of awareness about KC among the population in Aseer Province, Saudi Arabia. Additionally, a significant percentage of participants engaged in eye rubbing, a behavior associated with increased risk for KC. Itchiness, dryness, allergy, stress, and headache were all causes of eye rubbing. These results highlight the need for increased education and interventions through targeted educational campaigns and encouraging healthcare providers, particularly optometrists, and ophthalmologists, to routinely educate their patients about KC and the importance of avoiding eye rubbing. Further studies in other provinces of Saudi Arabia are needed to gain a deeper understanding and develop effective public health strategies.

## Appendices

**Table 5:** A list of questionnaire items

Tool 1: sociodemographic data	
A. Male	1. Gander:
B. Female	
A. 18-25	2. Age:
B. 26-35	
C. 36-45	
D. Older than 45	
A. Below secondary	3. Educational level:
B. Secondary	
C. University or higher	
A. Yes	4. Are you from Asir population:
B. No	
Tool 2: medical and family history of eye diseases among study participants	
A. Yes	1. Do you have any type of allergy?
B. No	
A. Chest allergy	2. If yes, what is the type of allergy? (multiple answers)
B. Skin allergy	
C. Eye allergy	
D. Nasal allergy	
E. Gastrointestinal tract allergy	
F. Others:…………..	
A. Yes	3. Have you had any visual or eyes disorder?
B. No	
A. Keratoconus	4. If yes, what was the disorder?(multiple answers)
B. Use visual lenses	
C. Amblyopia	
D. Refractive error	
E. Eye surgery	
F. Others:…………	
A. Yes	5. Have any one of your family members had keratoconus?
B. No	
Tool 3: awareness and perception of keratoconus and its relationship to eye rubbing	
A. Yes	1. Have you heard about keratoconus?
B. No	
A. Relative with keratoconus	2. If yes, what is the source of information regarding it?
B. Lectures/ reading	
C. social media	
D. Physician	
E. Friends	
A. Thinning of corneal thickness	3. What is keratoconus?
B. Increased corneal thickness	
C. Corneal inflammation	
D. I don’t know	
A. Yes	4. Is there any relationship between keratoconus and allergy?
B. No	
C. I don’t know	
A. Yes	5. Does keratoconus lead to visual impairment?
B. No	
C. I don’t know	
A. Surgery	6. What is the treatment method of keratoconus? (Multiple answers)
B. Medical glass	
C. Medical lenses	
D. Eye drops	
E. No treatment	
F. I don’t know	
A. A habit that may lead to keratoconus	7. What is frequent eye rubbing?
B. A habit that may harm the eye	
C. It may cause allergy/itch	
D. A safe habit	
E. I don’t know	
A. Yes	8. Do you rub your eyes frequently?
B. No	
A. Stress or headache	9. If yes, why do you rub your eyes?
B. Allergy	
C. Itching	
D. Dryness	
E. Others:………	
